# A comparative study on the effects of Mitchell and Benson relaxation techniques on quality of life of the old people in nursing homes: a quasi- experimental study

**DOI:** 10.1186/s12877-023-04378-z

**Published:** 2023-10-24

**Authors:** Aida Jasour, Ardashir Afrasiabifar, Mohammad Zoladl, Nazafarin Hosseini

**Affiliations:** 1 Student Research Committee, Yasuj University of Medical Sciences, Yasuj, Iran; 2 Department of Nursing, Yasuj University of Medical Sciences, Yasuj, Iran; 3 Social Determinants of Health Research Center, Yasuj University of Medical Sciences, Yasuj, Iran

**Keywords:** Benson Relaxation, Mitchell Relaxation, Oldest old, Quality of life, Nursing home

## Abstract

**Background:**

There is slight evidence on the effectiveness of relaxation techniques to improve quality of life of the old people, and no comparative studies have particularly investigated this population. Hence, the present study was conducted to examine the effect of Mitchell relaxation versus Benson relaxation technique to improve quality of life of the old people.

**Methods:**

In the present quasi-experimental study, 96 eligible old people in a nursing home were selected by available sampling method. Afterwards, they were assigned to three groups: Mitchell’s Relaxation Technique, Benson Relaxation Technique, and control (each of 32 participants) using the random block sampling method. The intervention groups received relaxation for 8 weeks and 3 sessions of 20 min each week. However, the control group did not receive any relaxation. Data was gathered by questionnaires (SF-36) and (CASP-19) before (week 0) and after the intervention (week 8) and were analyzed using the SPSS software version 26.

**Results:**

The results indicated that both Benson and Mitchell relaxation had improved the quality of life (SF-36) and (CASP-19) and their sub-scales in the participants compare to the control group (P < 0.001). Accordingly, the median (quartile 25, 75) of the specific quality of life of the participants before the intervention was 21 (18.25, 25.75) in the Benson group, 20.5 (16, 24) in the Michel group, and 21 (16.25, 24) in the control group. However, after the intervention they reached 35(26.25, 38.75), 34.5(26.75, 42.25), and 17 (14, 21) respectively. There was no statistically significant difference between the Benson and Michel relaxation groups.

**Conclusions:**

Based on the results, Benson and Mitchell relaxation techniques improve the quality of life of the old people. If the results be confirmed in other studies, the education of each of them, especially for the old people living in nursing homes and their caregivers, is suggested as routine care.

**Supplementary Information:**

The online version contains supplementary material available at 10.1186/s12877-023-04378-z.

## Introduction

According to the definition of the World Health Organization, people over 65 years old are considered old people [1]. According to statistics in 2015, the old population constituted about 15.3% of the world’s population, and it is predicted that it will increase to 20.8% in 2025 [2]. Aging is a natural process in which the physiological function of the body decreases and as a result, chronic clinical problems, physical movement, mental-cognitive and psychological diseases and some disorders occur, including depression, anxiety, sleep disorders [3]. These changes can lead to a decline in the quality of life (QOL) of the old people QOL is people’s satisfaction with daily life which includes physical, psychological, and social areas of life [1]. The increase of age and economic and social conflicts, physical and movement disorders and other problems of the older individual has led to their care to be transferred from home to nursing homes. As a result, their maintenance and care in nursing homes has been growing. Living in a nursing home can disrupt privacy, self-esteem, ability, and relationships with other people [4]. One out of six elder residents of nursing homes have anxiety and depression complications. All these problems affect their quality of life [5]. Thus, the old people who live in nursing homes are vulnerable and need more attention [6].

According to numerous studies, older individuals living in nursing homes have a lower quality of life than his/her peer who lives at home. Therefore, in addition to achieving a good quality of care [7], nursing homes should focus on improving the QOL of their residents [8]. Moreover, the aim of the elder people is not only to live longer, but also the quality of their life is important for them too [9]. Supporting or maintaining the quality of life of the elders is a social and moral obligation of governments and societies that should be given special attention [10].

In order to improve the QOL of the old people, various interventions and health care, including health interventions, interdisciplinary interventions and sports interventions, have been used which have had different outcomes [11]. Consistent physical activity strengthens the immune system and can improve psychological health by reducing anxiety and depression and increasing self-confidence [12]. Amassed muscle and joint disorders in the older individual can prevent exercise. As a result, the use of active relaxation techniques which do not require muscle involvement has received more attention [13]. One of these methods is the Benson’s relaxation technique (BRT). This method was introduced by Benson, which consists of mindfulness techniques [14]. BRT has a major effect on reducing anxiety, depression, and improving QOL and psychological factors. Furthermore, this technique is easy to learn and no side effects have been reported [15].

Mitchell’s Relaxation Technique (MRT) is another relaxation technique. The basis of this method is mutual inhibition of muscles and diaphragmatic breathing as well; i.e., when a muscle is involved, the muscles of the opposite group are in a state of relaxation [16]. MRT is an auditory relaxation method which focuses on the psycho-immunological connection between mind and body and comprises guided imagery and relaxation, breathing, and muscle exercises [17]. Studies have displayed that MRT has significant effects in improving immune function, reducing depression, stress, anxiety, pain and improving quality of life [18]. In addition, MRT is a highly effective, non-invasive, safe, cheap and easy-to-implement method, which has led to more focus on this method [19].

In a study, Biabani et al. found Jacobson method to be more effective than BRT on elderly depression [19]. In a study, Elsayed et al., indicated the high effect of BRT on improving anxiety, depression and sleep quality in the old people undergoing hemodialysis [15]. However, no study was found regarding the effect of BRT and MRT single-handedly on the QOL of the old person and comparing the effects of these two methods on the QOL of the older individual.

Isolation problems, lack of family support and presence with the old people, disability, physical and psychological problems such as depression, stress and anxiety have reduced the QOL of the old people living in nursing homes. On the other hand, the side effects of drugs in old age and the limitation of physical activity of the old people make it necessary to investigate the effect of palliative care, including Benson and Michel’s relaxation techniques, and to find the best technique for improving their quality of life. Therefore, the present study was conducted with the aim of comparing the effects of Benson’s Relaxation Technique and Mitchell’s Relaxation Technique on the quality of life of the old people living in nursing homes. Moreover, this question is raised that which of these two relaxation methods has a greater effect on the quality of life of the residents of nursing homes?

## Methods

### Study design

The present research was a quasi-experimental controlled study with a pre-test post-test design. The interventions included both Benson and Mitchell the relaxation techniques, and the outcome variable was the quality of life of the old people, which was measured before (week 0) and after the intervention (week 8).

### Participants and sampling

The study population included the old people residents of Khwaja Nasir al-Din Toosi Nursing Home in Maragheh, Mohabbate in Tabriz and Alavi Azarshahr in East Azarbaijan Province, Iran. The research location was Khwaja Nasir al-Din Tousi’s nursing home Mohabbate and Alavi, which are respectively located in the cities of Maragheh, Azarshahr and Tabriz in East Azarbaijan Province, Iran.

The number of samples was based on the parameters α = 0.05, α-1=/95, Β = 0.2, β=-0.85, Z1 -α/2 = 1.96 and Z1 -β = 85/85. Moreover, the standard deviation of the quality of life was equal to µ1 = 5.1, 1.34 and µ2 = 30.2, 14, the number of people in each group was 26. However, taking into account 20% attrition, a total number of 32 people in each group and a total of 96 people were considered [5].

Eligible participants were selected by available sampling method and were assigned to 3 groups of MRT (32 people), BRT (32 people) and control group (32 people) using random block sampling method.

First, by calculating the number of study groups (two intervention groups and one control group), the number of people in each block was equal to 3, and then using the factorial law (3! =1 × 2 × 3 = 6) the number of 6 blocks were calculated. The randomization and selection of blocks continued until 32 elder individuals were placed in the Benson relaxation intervention group, 32 older persons were placed in the Mitchell relaxation intervention group, and 32 older individuals were placed in the control group.

Inclusion criteria included: ability to communicate and answer questionnaire questions, age over 65 years old, living in a nursing home, obtaining a lower-than-average QOL score (28–78) and cooperating with the researcher to carry out interventions. Exclusion criteria were mental or cognitive impairment in according to medical records, and physical disability. In addition, those who did not meet the inclusion criteria were excluded from the study.

### Measurement

The data were gathered by two structured and self-reported questionnaires, including the 36-Item Short Form Survey of quality of life (SF-36 QOL) and the special questionnaire for measuring the quality of life of the old people including constructs of ‘control’, ‘autonomy’, ‘self-realization’, and ‘pleasure’ (CASP-19 QOL).

SF-36 QOL was developed by Ware and Sherbourne [20]. This questionnaire included 36 statements and 8 subscales including physical functioning (items 10 items), social functioning (2 items), role limitations due to physical problem (4 items), role limitation due to emotional problem (3 items), perception of mental health (5 items), vitality (4 items), bodily pain (items 2 items) and general health (5 items). The subject’s score in each of these subscales ranges from 0 to 100. A higher score means a better quality of life. The validity and reliability of the questionnaire in the Iranian population was confirmed by Montazeri et al. [21, 22]. With the Cronbach’s α coefficients ranging from 0.77 to 0.90 exception of the vitality scale (α = 0.65), and Convergent validity above 0.40 ranging from 0.58 to 0.95.

The CASP-19 QOL was designed in England. It includes 19 specific items that consist of 4 subscales of control (4 items), autonomy (5 items), self-realization (5 items), and pleasure (5 items). The scales of this questionnaire are Likert ratings, which have four options: “most of the time”, “sometimes”, “not often” to “never”. The lowest score for each statement is “zero” and the highest “score” is “three”. The scoring of the 6 statements of the questionnaire includes items 8, 6, 4, 2, 1, and 9 were scored in a reverse form [23].

This questionnaire has been translated into different languages. In Iran, Heravi et al. confirmed its validity and reliability in the Iranian society in 2014. The maximum score of the questionnaire is 57 (full satisfaction from all 4 dimensions) and the lowest is zero (lack of quality of life) [24, 25].

Considering that Some of the participants were illiterate and to avoid bias, the questionnaires were completed by structured interview with all the participants by a colleague at the beginning of the study (week 0) and at the end of the interventions (week 8).

### Procedures

#### Intervention group 1

All sessions of Mitchell relaxation intervention were completed by the participants for 8 weeks and every week during 3 sessions of 20 min (total 24 sessions) under the guidance of two researchers (community health nurses) according to the relaxation protocol Mitchell [17].

#### Intervention group 2

The Benson relaxation intervention was completed by the elder individual for 8 weeks and every week during 3 sessions of 20 min (total 24 sessions) under the guidance of the researcher (community health nurse) in a room in order to respect the privacy of the participant according to Mitchell relaxation protocol [26, 27].

The intervention for each participant was done separately in a room at the nursing home respecting the privacy of the patient under supervision and guidance of the researcher. All intervention sessions at Khwaja Nasir al-Din Toosi Nursing Home in Maragheh were conducted on even days and in Alavi Nursing Home in Azarshahr in East Azarbaijan Province, and Mohabbate in Tabriz in Iran on odd days from 8:00 am to 16:00 pm under the supervision and guidance of the researcher and a colleague (community health nurses holding MSc trained by a psychiatry nurse as a member of the research team) and were implemented by the participants. In order to avoid bias in each nursing home, based on the fixed list of participants, odd numbers were assigned to the researcher and even numbers assigned to the colleague, and vice versa for the next session.

#### Control group

The control group did not receive any relaxation technique.

### Data analysis

Data were analyzed using the SPSS software (version 26) with a significance level of P < 0.05. One-way analysis of variance test was used for the age variable, and chi-square test was used for other demographic information. The data distribution was checked using the Kolmogorov-Smirnov test, the results indicated that they do not have a normal distribution. Therefore, Kruskal-Wallis tests were used for between-group comparisons, Bonferroni post hoc tests for pairwise comparisons, and Wilcoxon tests for intra-group comparisons.

## Results

**Fig. 1:**
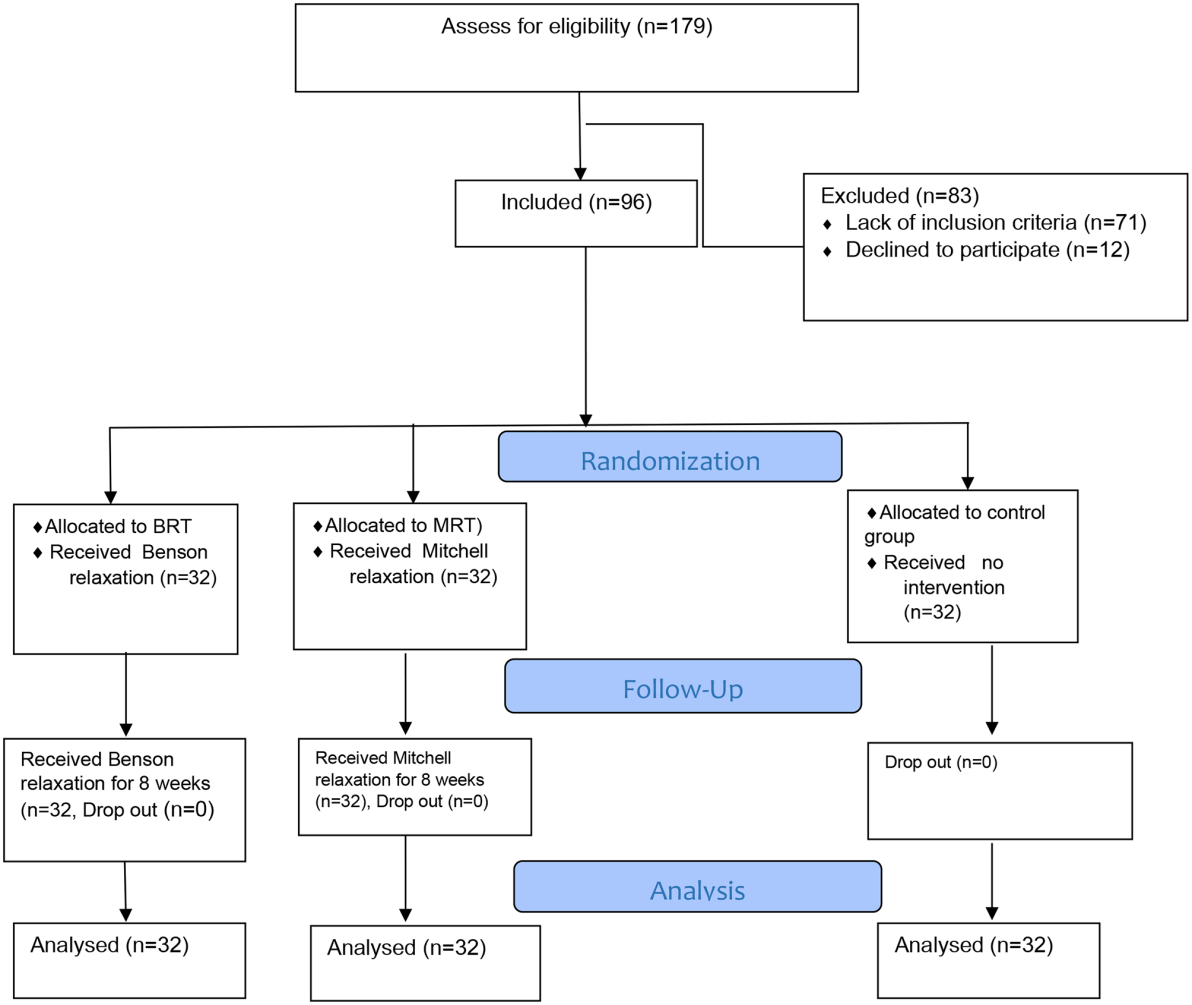
CONSORT Diagram for the study

In the present study, 96 participants remained until the end of the study (Fig. 1). The average age of the older individual in the three groups was 70.5 ± 8.7 years. There was no significant statistical difference between the three groups in the variables of age, gender, education and occupation (Table 1).

**Table 1:** Demographic characteristics of older person

Group Variables		Benson (n = 32)	Mitchell (n = 32)	Control (n = 32)	p- value
Age; M(SD)		70.8(8.3)	68.8(6.9)	72.3(8.1)	0.207*
Gender;n (%)	Male	22(68.7)	17(53.1)	21(65.6)	0.393**
	Female	10(31.3)	15(46.9)	11(34.4)	
Job : n (%)	Unemployed	17(53.1)	16(50)	14(43.8)	0.391**
	Employed	15(46.9)	16(50)	18(56.2)	
Education level:n (%)	Illiterate	16(50)	14(43.8)	17(53.2)	0.475**
	Less than Diploma	14(43.8)	13(40.6)	14(43.7)	
	Diploma & Greater	2(6.2)	5(15.6)	1(3.1)	

M(SD): Mean (Standard Deviation); * One- Way ANOVA; **Chi-Square test

Before the research intervention, no statistically significant difference was observed in the distribution of the QOL (CASP-19 questionnaires), and the QOL (SF36) of the participants and their subscales, between the intervention and control groups. After the intervention, however, a statistically significant improvement was observed in the distribution of the QOL (CASP-19 questionnaires) and the QOL (SF36) of the participants and their subscales, between the intervention and control groups (P < 0.05) (Tables 2 and 3).

**Table 2:** Between and within group comparison for median scores of Specific QOL (CASP- 19)

Variables	Group Time	Benson Relaxation	Mitchell Relaxation	Control	Between group Comparison
		Median (Q1,Q3)	Median (Q1,Q3)	Median (Q1,Q3)	*p-value
Quality of Life (CASP- 19)	Pre test	21(18.25,25.75)	20.5 (16, 24)	21 (16.25, 24)	0.595
	post test	35(26.25, 38.75)	34.5(26.75, 42.25)	17 (14, 21)	0.001
	**Within group Comparison	0.002	0.001	0.001	
Autonomy	Pre test	6 (4, 8)	6 (4, 7)	7(4, 7.75)	0.437
	post test	8 (6,10)	9 (6.5,11)	7(4, 7.75)	0.001
	**Within group Comparison	0.002	0.001	0.037	
Self-realization	Pre test	4 (3, 5)	5 (3.25, 6)	4 (3, 5)	0.169
	post test	5 (2.25, 5)	10.5 (6.25, 12.75)	5 (2.25, 5)	0.001
	**Within group Comparison	0.001	0.001	0.914	
Pleasure	Pre test	5 (3, 8)	5.5 (3, 8)	5 (3, 8)	0.812
	post test	4.5 (3,6)	9 (6.25, 11)	4.5 (3, 6)	0.001
	**Within group Comparison	0. 001	0.001	0073	
Control	Pre test	4 (4,6)	4 (4,6)	4 (4,6)	0.312
	post test	4 (2,6)	7 (5,7)	4 (2,6)	0.001
	**Within group Comparison	0.016	0.001	0.057	

Median (Q1, Q3): Median (Quartile 25, Quartile 75), *Kruskal- Wallis test;** Wilcoxon test

**Table 3:** Between group comparison for median scores of Specific QOL (SF36)

Variables	Group Time	Benson Relaxation	Mitchell Relaxation	Control	Between group Comparison
		Median (Q1,Q3)	Median (Q1,Q3)	Median (Q1,Q3)	*P- value
quality of life (SF-36)	Pre test	41.24 (34,26, 49.95)	42.72 (33.01, 52.28)	40.92 (33.9,52.5)	0.993
	post test	60.85 (53.71, 77.5)	67.5 (53.42, 83,21)	41.43 (32.21, 49.16)	0.001
General health	Pre test	54(37,66)	47.9 (30.19, 54.14)	54.1 (37.5,66.6)	0.177
	post test	50 (33,60)	75.5 (62.5, 86.45)	50 (33.3, 66)	0.001
Physical functioning	Pre test	25 (20,40)	40 (21.25, 50)	25 (20,40)	0.310
	post test	50 (26,70)	55 (21.25, 78.75)	25 (15, 38.75)	0.001
Role-physical	Pre test	0(0,25)	25 (0,25)	10 (0,25)	0.294
	post test	25 (0,50	50 (0, 75)	0(0, 25)	0.002
Emotional functioning	Pre test	0(0, 33.3)	0(0, 33.3)	33.3(0, 33.3)	0.054
	post test	33.3 (33.3, 66,6)	33.3 (33.3,100)	33.3 (0, 33.3)	0. 001
Social functioning	Pre test	50 (37.5,50)	50 (37.5, 62.5)	50 (37.5, 50)	0.092
	post test	65 (50,87, 5)	62.5 (50, 87.5)	37.5 (37.5, 50)	0. 001
Bodily pain	Pre test	55(35,76.87)	50(35.62, 65)	45(35, 67.5)	0.241
	post test	83.75 (54.25, 100)	77.5 (49.37, 97.5)	41.25 (25, 67.5)	0. 001
Vitality (fatigue/happiness)	Pre test	40(30,50)	40(31.25, 50)	45(30, 52.5)	0.681
	post test	65(50, 75)	60(50,79)	35(22.5, 50)	0.0001
Mental health	Pre test	44(36, 51)	45(32.75, 52)	48(33, 58)	0.779
	post test	64(52, 76)	68(56, 80)	44(30, 48)	0. 001

Median (Q1, Q3): Median (Quartile 25, Quartile 75), *Kruskal- Wallis test

In a pairwise comparison, a significant difference was displayed in the distribution of the QOL (CASP-19 questionnaires), and the QOL (SF36) of the participants and their subscales between each of the Benson and Mitchell intervention groups compared to the control group (p < 0.0001). On the other hand, no statistically significant difference was observed in the distribution of both QOL scores and their subscales between the Benson and Mitchell relaxation groups.

The distribution of all subscales of the QOL (CASP-19 questionnaires), and their subscales in the Benson and Mitchell relaxation intervention groups indicated a significant improvement after the intervention compared to beforehand (p < 0.05). In the control group, there were a significant decrease in the distribution of the QOL (CASP-19 questionnaires) of the participants (p = 0.001) and its autonomy dimension (p = 0.037) after the research intervention compared to before. (Table 2).

The results indicated that the distribution of quality of life (SF36) and its subscales of the participants in the two intervention groups of Benson and Mitchell intervention compared to before the intervention indicated a statistically significant improvement (p < 0.05). On the other hand, in the control group, only the vitality subscale distribution exposed a significant decrease after the research intervention compared to before the intervention. (Table 4).

**Table 4:** Within group comparison for median scores of Specific QOL (SF36)

Variables	Group	Pre test	Post test	P-value
		Median (Q1,Q3)	Median (Q1,Q3)	
quality of life (SF-36)	Benson	41.24(34,26,49.95)	60.85(53.71, 77.5)	0.001
	Mitchell	42.72(33.01,52.28)	67.5(53.42, 83,21)	0.001
	control	40.92(33.9,52.5)	41.43(32.21,49.16)	0.052
General health	Benson	54(37,66)	50(33,60)	0.001
	Mitchell	47.9(30.19,54.14)	75.5(62.5, 86.45)	0.001
	control	54.1(37.5,66.6)	50(33.3,66)	0.097
Physical functioning	Benson	25 (20,40)	50(26,70)	0.001
	Mitchell	40 (21.25, 50)	55(21.25,78.75)	0.001
	control	25 (20,40)	25(20,40)	0.091
Role- Physical	Benson	0(0, 25)	25(0,50)	0.001
	Mitchell	25(0, 25)	50(0,75)	0.001
	control	10(0,25)	0(0,25)	0.655
Emotional functioning	Benson	0(0,33.3)	33.3(33.3,66.6)	0.001
	Mitchell	0(0,33.3)	33.3(33.3, 100)	0.001
	control	33.3(0,33.3)	33.3(0,33.3)	0.222
Social functioning	Benson	50(37.5,50)	65(50,87,5)	0.001
	Mitchell	50(37.5, 62.5)	62.5(50,87.5)	0.001
	control	50(37.5,50)	37.5(37.5,50)	0.726
Bodily pain	Benson	55(35,76.87	83.75(54.25, 100)	0.001
	Mitchell	50(35.62, 65)	77.5(49.37, 97.5)	0.001
	control	45(35, 67.5)	41.25(25,67.5)	0.298
Vitality (fatigue/happiness)	Benson	40(30,50)	65(50,75)	0.001
	Mitchell	40(31.25, 50)	60(50,79)	0.001
	control	45(30,52.5)	35(22.5,50)	0.029
Mental health	Benson	44(36,51)	64(52,76)	0.001
	Mitchell	45(32.75, 52)	68(56,80)	0.001
	control	48(33,58)	44(30,48)	0.137

Median (Q1, Q3): Median (Quartile 25, Quartile 75), *Wilcoxon

## Discussion

The present study compared the effect of BRT and MRT on the QOL of the elderly residents of nursing homes. To date, no study parallel to the present study has been conducted. The results of the present study indicated that the implementation of MRT and BRT in the intervention groups had improved the QOL (CASP-19), its subscales, the QOL (SF-36) and its subscales (general health, physical function, role limitation due to physical problem, limitation due to physical problem, social function, bodily pain, vitality, and perception of mental health) of the participants. On the other hand, both BRT and MRT had improved quality of life scores (SF-36) and specific quality of life of the participants and their subscales.

In the present study, the Benson relaxation intervention improved the QOL (CASP-19) and QoL (SF-36) and most of their subscales. Similar to the results of the present study, in the study by Habibollahpour et al., it was revealed that the quality of sleep of the old people using Benson’s relaxation method had improved [28]. In the mentioned study, the outcome was sleep and Benson relaxation was performed daily for 4 weeks, but in the present study, it was performed 3 times a week for 8 weeks. However, in both studies, Benson’s intervention indicated improved outcomes. In addition, in a study by Haruni et al., Benson intervention improved anorexia and sleep quality in cancer patients undergoing chemotherapy [29]. Based on the above study, the BRT had improved the quality and duration of sleep. Although the outcome variables in the mentioned studies and the present study are different, it can be understood that improving sleep by using relaxation techniques in the old individuals can lead to the improvement of their quality of life.

In line with the findings of the present study, the results of a systematic review by Pangaribuan et al. exhibited that the BRT reduced depression, stress, and anxiety in hemodialysis patients and improved their quality of life and mental health [30]. In the mentioned study, only two studies had investigated the effect of Benson’s relaxation technique on the quality of life, i.e., relaxation was performed once a day for 8 weeks in hemodialysis patients aged 18–67, but in the present study, this intervention was performed 3 times a week on the participants for 8 weeks. However, in both studies, Benson’s technique improved the QOL of the participants. Similar to the results of the present study, the positive effect of Benson relaxation in improving patients’ anxiety in emergency patients was observed in studies by Ibrahim et al., and reducing anxiety before surgery in candidates for open heart surgery [31] was as well observed in a study by Malmir [32]. Ibrahim et al. used BRT with only one session in in non-elderly hospitalized patients in the emergency ward and in Malmir’s study, non-elderly open heart surgery candidates received BRT during 2 sessions before surgery, but in the two-studies anxiety decreased in the patients. On the other hand, in the research of Goudarzi et al., BRT reduced the anxiety of patients undergoing radial angiography [33]. In the mentioned studies, BRT was performed on the patients, which relieved their anxiety. It can be assumed that reducing anxiety as a result of Benson’s intervention can improve the mental health and thus the QOL of the old people. Additionally, in a study by Wulansari et al., Benson relaxation combined with mental exercises improved depression in the old people [34]. In the mentioned study, the old participants received brain gymnastics therapy and BRT for 11 sessions which eventually reduced their depression. Decreasing depression in the old people can as well help improve their mental health, as in the present study, Benson improved the subscale of mental health in the old people. Benson relaxation stimulates the release of calcium by regulating the levels of neurotransmitters, thereby increasing the release of dopamine and acetylcholine. These factors are effective for maintaining nervous functions, fostering positive mood and increasing cognitive performance [35].

In the present study, Mitchell relaxation intervention improved the QOL (CASP-19) of the participants and the QOL (SF-36) and its subscales. Similar to the results of the present study, Amirova et al., indicated that the Mitchell relaxation intervention was effective in reducing pain, fatigue, and sleep problems and improved the QOL of the patients with fibromyalgia [18]. Relaxation methods are used to relieve muscle pressure, and as a result, reduce physical pain, improve physical performance and mental health, and as a result, improve the quality of life. Performing relaxation exercises improve an individual’s sense of health by stabilizing the autonomic nervous system and controlling one’s emotions in tense and stressful situations. Relaxation is the establishment of a state of general relaxation, which is the opposite of tension, stress, and depression. Correspondingly, relaxation methods affect the body’s physiology and the patient’s sympathetic system by reducing the body’s metabolism, the number and strength of heart contractions, respiration rate, epinephrine secretion, and blood pressure.

The results of the present study indicated that both Mitchell and Benson relaxation techniques improved the QOL of the participants. A study comparing the effect of Benson and Mitchell relaxation techniques was not found, but there were comparisons between each of these methods with other relaxation techniques. In a research by Moradi et al., Jacobson’s technique was more effective than Benson’s on depression in the older individual [36]. This superiority of Jacobson’s technique over Benson’s can be due to the fact that Jacobson’s technique is active and the old people were more involved in Benson’s relaxation intervention. On the other hand, in a study by Ganesh et al., the effectiveness of MRT on improving pain and the effectiveness of Jacobson relaxation technique on improving the quality of life of people with primary dysmenorrhea were shown, although, both relaxation techniques, similar to the present study, improved the quality of life of women. Moreover, in both interventions, the average menstrual distress improved, although, no significant difference was observed [17]. Similar to the present study, MRT is considered to be effective in improving subscale of mental health in quality of life of the participants. In the mentioned study, each intervention was performed twice a day for two consecutive menstrual cycles in women with primary dysmenorrhea. whereas in the present study the old people received 24 intervention sessions for 8 weeks. In addition, the sample size of the study was smaller than of the present study.

Contrary to the results of the present study, a study by Hoseinian et al., Jacobsen’s relaxation technique using a mobile application did not improve the anxiety and occupational stress of nurses caring for patients with Covid-19 [37]. However, in the present study, Benson and Jacobson’s relaxation techniques improved the quality of life of the old people even during the COVID-19 pandemic. Perhaps the cause of this difference was the use of the virtual application by the participants, which was not supervised for the techniques as in the present study. However, in the present study, the interventions were implemented under the supervision and management of the researcher.

In the present study, the independence dimension of the QOL (CASP-19) in the control group declined after the research intervention, which was expected from the old people residing at nursing homes. The researcher’s control over the environment and time of the research, supervision and assistance of the researcher in carrying out interventions by the old people and no exclusion of participants in the research period were among the strengths of the present research.

The limitations of the present study were the lack of follow-up time after the intervention and the completion of two QOL questionnaires with many questions, which due to the age and complications of the old people individuals may have bored them that may have led them to answer the questions with less care.

## Conclusion

Based on the results of the present study, it can be assumed that the use of each of the Benson and Mitchell relaxation techniques improved the QOL of the old people. Therefore, the instruction of each of the two relaxation methods is suggested as effective, comfortable and low-cost methods for the older individuals, especially the old people living in nursing homes and their caregivers as routine care.

In addition, the use of both the specific quality of life (CASP-19) and the quality of life (SF-36) questionnaires have displayed similar results. Therefore, only one questionnaire, preferably a specific quality of life questionnaire, should be used to measure the quality of life of the old people.

It is suggested that the effect of relaxation interventions on the quality of life be investigated in a follow-up period of 3 to 6 months.

### Electronic supplementary material

Below is the link to the electronic supplementary material.

Supplementary Material 1

## Data Availability

The datasets generated and/or analyzed during the current study are not publicly available but are available from the corresponding author on reasonable request.

## Abbreviations

QOL: Quality of Life
BRT: Benson’s relaxation technique
MRT: Mitchell’s Relaxation Technique
SF-36: The 36-Item Short Form Survey
CASP-19: Control, autonomy, self-realization, and pleasure
