# Supplementary material for: A comparative study on the effects of Mitchell and Benson relaxation techniques on quality of life of the old people in nursing homes: a quasi- experimental study
Source: BMC Geriatr. 2023 Oct 24;23:692. doi: 10.1186/s12877-023-04378-z (PMC10598898; doi:10.1186/s12877-023-04378-z)
Supplement: Supplementary file 1 — Supplementary Material 1 [file 12877_2023_4378_MOESM1_ESM.docx]

**Mitchell relaxation intervention protocol**

1. The older individual was moved away from the stressful situation. 2. He/She was placed in a calm and quiet room. Then the elderly was told to: 3. Pull his/her jaw down and then stop , and get into the new position. 4. The tongue should be pressed downwards in the mouth, so that he/she feels this step. 5. If he/she opened his/her eyes, he/she was told to slowly close his eyes, keeping his eyelids down, then he stops and the person is asked to enjoy the peace and darkness. 6. Push him/her head down, opposite to the bed, then stops this action and feels relief from the weight of the head and that the bed carries the weight of the head. 7. Pull oneself shoulders towards oneself feet and feel the space between shoulders and ears, then stop this action and feel the new position. 8. Move oneself elbows to the side away from their body until they would reach a comfortable point. 9. Then he/she was asked to stop moving and feel the space between his/her arms and body. 10. Breathe slowly and deeply, without any effort or change in breathing rhythm. 11. Turn oneself thighs outward (external rotation) and stop in this position. Place oneself feet in a comfortable position and understand and feel the new position. 12. Move oneself knees to feel comfortable, adjust oneself positions to increase comfort. 13. The feet should be carefully moved away from the body, so as not to suffer from muscle cramps and to feel the new position. 14. Push oneself body towards the bed and feel the bearing of oneself weight and then feel all the parts of body that are in contact with the bed. 15. Thinks to a smooth movement that started from above the person's eyes. Moves towards the hairline, continue to the top of the crown and down the neck and feels the pleasure and comfort of this movement. The person was then told to gradually return to the active position to avoid fainting. He/She was asked to open his eyes, to be aware of the room, and give gentle stretching to the limbs. The body needs some time to return to an active state.

**Benson relaxation intervention protocol**

The older person was told to: 1. Slowly get into a comfortable position. 2. Close the eyes slowly. 3. Keeps all the muscles of the body from the soles of the feet to the face slowly loosed and relaxed. 4. Breathe through the nose and be aware of your breathing. Breathe out slowly through the mouth, when the breath comes out, repeat the number one under the lips and breathe easily and normally. 5. Does this for 15 minutes and tries to relax the muscles. Then slowly open their eyes and don't get up for a few minutes. 6. should not worry about whether he/she had reached a deep level of relaxation or not, lets the relaxation happen with its own song. When disturbing thoughts occur, tries to ignore them and be indifferent to them.
